# COVID-19 vaccination induces cross-reactive dengue virus antibodies with altered isotype profiles and *in vitro* antibody-dependent enhancement

**DOI:** 10.3389/fimmu.2025.1683070

**Published:** 2025-12-17

**Authors:** Sebastian Reinig, Chin Kuo, Sheng-Yu Huang, Kuei-Ching Hsiung, Po-Kai Chen, Etsuro Ito, Ing-Kit Lee, Ching-Yen Tsai, Shu-Min Lin, Shin-Ru Shih

**Affiliations:** 1 Research Center for Emerging Viral Infections, Chang Gung University, Taoyuan, Taiwan; 2 Biomedical Translation Research Center and Institute of Biomedical Sciences, Academia Sinica, Taipei, Taiwan; 3 Department of Biology, Waseda University, Tokyo, Japan; 4 Graduate Institute of Medicine, Kaohsiung Medical University, Kaohsiung, Taiwan; 5 R&D Department, BioPhenoMA Inc., Tokyo, Japan; 6 Division of Infectious Diseases, Department of Internal Medicine, Kaohsiung Chang Gung Memorial Hospital, Kaohsiung, Taiwan; 7 College of Medicine, Chang Gung University, Taoyuan, Taiwan; 8 Division of Infectious Disease, Department of Internal Medicine, Kaohsiung Municipal Feng-Shan Hospital, Kaohsiung, Taiwan; 9 Department of Thoracic Medicine, Chang Gung Memorial Hospital, Chang Gung University, School of Medicine, Taoyuan, Taiwan; 10 Department of Respiratory Therapy, Chang Gung Memorial Hospital, Chang Gung University, School of Medicine, Taoyuan, Taiwan

**Keywords:** dengue, isotype, cross-reactive, antibody-dependent enhancement, COVID-19

## Abstract

**Background:**

SARS-CoV-2 infection and COVID-19 vaccination can induce antibodies that cross-react with dengue virus (DENV). Pre-existing IgG antibodies against DENV are known to enhance infection through antibody-dependent enhancement (ADE) via Fcγ receptors. COVID-19 vaccines induce an altered IgG subclass distribution with modified Fcγ receptor affinity. This study investigates the isotype profile of DENV cross-reactive antibodies induced by COVID-19 vaccination and their potential to mediate ADE.

**Methods:**

We retrospectively collected 271 serum samples from individuals in Taiwan who were either SARS-CoV-2-unvaccinated, vaccinated with 1–3 doses of COVID-19 vaccine, recipients of an Omicron-era booster, or previously recovered from dengue infection. Antibody titers (IgM, IgA, and IgG subclasses 1–4) against SARS-CoV-2 spike (S) glycoprotein and DENV serotype 2 envelope (E) protein were measured by enzyme-linked immunosorbent assay (ELISA). Selective depletion of anti-S or anti-E antibodies was performed to confirm cross-reactivity. ADE activity was assessed *in vitro* by incubating DENV-2 particles with serum antibodies followed by infection of Fcγ receptor-bearing THP-1 monocytic cells.

**Results:**

Cross-reactive anti-E antibodies in COVID-19-vaccinated individuals were predominantly IgM and IgA, whereas anti-S antibodies in vaccinated individuals and anti-E antibodies in dengue-recovered patients were dominated by IgG1. Low levels of cross-reactive anti-E IgG detected in vaccinated individuals were mainly of the low-affinity IgG2 and IgG4 subclasses. These vaccine-induced cross-reactive antibodies mediated significantly stronger *in vitro* ADE compared with antibodies from natural DENV infection, particularly after the first or second vaccine dose and after Omicron-variant boosters. Inactivation of complement reduced ADE in COVID-19-vaccinated sera but enhanced it in sera from dengue-recovered individuals.

**Conclusion:**

COVID-19 vaccination induces DENV cross-reactive antibodies with a distinct isotype profile and distinct ADE compared with natural dengue infection. Due to low Fc-affinity these antibodies likely do not pose a threat of dengue. These findings highlight the importance of considering off-target immunity and ADE risk in future vaccine design.

## Introduction

Dengue virus (DENV) infection poses a significant public health challenge in tropical and subtropical regions, with over 400 million cases and approximately 20,000 deaths annually. This *Flavivirus* is transmitted by mosquito vectors. While most infections are mild or asymptomatic, a small proportion can progress to severe conditions, such as dengue hemorrhagic fever or dengue shock syndrome (1). Although adaptive immunity typically provides protection, a secondary dengue infection can increase disease severity in some individuals. This is believed to be due to antibody-dependent enhancement (ADE). In ADE, IgG antibodies bind to DENV, promoting its entry into mononuclear immune cells through Fc receptors and potentially intensifying macrophage inflammation (2, 3). The structural envelope protein (E-protein) of DENV cross-reacts with antibodies elicited by other viruses, particularly other flaviviruses (4). Cross-reactivity between SARS-CoV-2 spike glycoprotein (S-protein) and DENV E protein was first reported in false positives of COVID-19 recovered in dengue anti-E rapid tests (5). Cross-reactive epitopes have since been identified, including monoclonal antibodies against dengue and polyclonal antibodies from COVID-19 patients (6–8). SARS-CoV-2 mRNA and protein subunit vaccines induce a gradual shift in antibody subclasses, transitioning from high Fc-receptor affinity IgG1/IgG3 to low Fc-receptor affinity IgG2/IgG4 over time (9–11). Previous studies have demonstrated that IgG2/IgG4 antibodies, despite sharing the same antigen-binding Fab region, exhibit a lower Fc-receptor affinity and reduced maximum *in vitro* ADE capacity for dengue than those of other subclasses (12). Additionally, an elevated ratio of anti-E IgG1 to IgG2 antibodies is linked to increased disease severity in dengue (13). Afucosylated anti-dengue antibodies, which exhibit enhanced binding affinity for the FcγIIIa receptor, have been associated with severe disease and heightened infection risk (3, 13–16). Thus, the Fc-structure of IgG is critical for ADE potential in dengue. However, it is not clear whether COVID-19 vaccinations affect the isotype distribution of cross-reactive antibodies and their potential to induce ADE. To address this issue, we compared the isotypes of anti-dengue antibodies in individuals vaccinated against COVID-19 and those who recovered from DENV infection. Additionally, we evaluated the cross-reactivity of these antibodies and their capacity to induce ADE.

## Material and methods

### Sample collection

In total, 271 serum or plasma samples were collected from individuals before vaccination and after the first, second, and third doses of the following vaccines: the adenovector vaccine ChAdOx1 nCoV-19 (AstraZeneca, AZ, Cambridge, UK); the protein subunit vaccine MVC-COV1901 (Medigen, Frederick, MD, USA); and the mRNA vaccines mRNA-1273 (Moderna, Norwood, MA, USA) and BNT162b2 (BioNTech/Pfizer, Mainz, Germany) (**Table 1**). Furthermore, 21 samples were collected from individuals who received an Omicron targeted-booster: Moderna (XBB), Novavax (XBB), and Moderna (Ba.4.5) (**Supplementary Table S1**). Additionally, 24 samples from dengue convalescent individuals were collected at Kaohsiung Chang Gung Memorial Hospital or Kaohsiung Municipal Fengshan Hospital between February 10 and December 7, 2023, following confirmation of DENV infection by PCR or ELISA through the hospital’s clinical laboratory (**Supplementary Table S2**). Serotypes were determined through a serum neutralization assay and identified in 13 of 24 samples; 10 were identified as DENV serotype 1 (DENV-1), 2 as serotype 2 (DENV-2), and 1 sample showed a mixed infection with both DENV-1 and DENV-2. The samples from Taiwan were collected from individuals at Chang Gung Memorial Hospital and Chang Gung University (ethics approval number: 202001041A3C). Negative human serum samples from the United States were purchased from Access Biologicals (**Supplementary Table S3**). These samples were collected as per protocol SDP-003, Human Biological Specimens Collection, dated September 22, 2017, and the qualifications of the principal investigator (Robert Pyrtle, M.D.) were reviewed and approved by the Diagnostics Investigational Review Board (Cummaquid, MA, USA).

**Table 1:** Age and sample collection timing (in days post-infection or symptom onset) for each group, reported as mean, minimum, and maximum values.

Variable	Unvaccinated (0)	Dose 1 (1)	Dose 2 (2)	Dose 3 (3)	Omicron-booster (omi)	Dengue recovered (DENV)
n	14	44	60	90	21	24
Age (years)	37.2 (21–59)	37.4 (21–74)	36.7 (21–60)	38.7 (20–62)	38.9 (25–62)	56 (22–83)
Female (%)	71.4	68.2	61.6	63.3	57.8	70.8
Vaccine	–	Moderna (6) BNT (10) Medigen (18) Astrazeneca (10)	Moderna (15) BNT (15) Medigen (18) Astrazeneca (12)	Moderna (21) BNT (14) Medigen (17) Astrazene-ca (1) Astrazeneca-heterolog (19) Medigen-heterolog (16)	Novavax-XBB (14) Moderna-XBB (5) Moderna Ba.4.5 (2) (**Supplementary Table S1**)	ND (13), Moderna (3), BNT-Moderna (1), Medigen (1), AZ-Moderna heterolog (2), (**Supplementary Table S2**)

Patients received ChAdOx1 nCoV-19 (AstraZeneca, AZ), MVC-COV1901 (Medigen), or the mRNA vaccines mRNA-1273 (Moderna) or BNT162b2 (BNT), ND: not determined.

#### Dengue virus serum neutralization assay

The DENV serum neutralization assay was performed using Vero cells. Falcon 96-well cell culture plates were used for cell seeding, and GeneDirex 96-well plates (Las Vegas, NV, USA) were designated for serum dilution and incubation. Prior to use, all serum samples were heat-inactivated at 56°C for 30 min.

Vero cells were cultured in Minimum Essential Medium (MEM) supplemented with 10% fetal bovine serum (FBS), 2% penicillin-streptomycin (P/S), 1% non-essential amino acids (NEAA), and 1% sodium pyruvate. For virus dilution and infection, a modified medium consisting of MEM with 2% FBS, 2% P/S, 1% NEAA, and 1% sodium pyruvate was used. The overlay medium was prepared by supplementing this modified medium with 1% methylcellulose.

To initiate the assay, Vero cells were seeded at a density of 2.5 × 10^4^ cells in 100 µL per well and incubated overnight at 37 °C in a 5% CO_2_ atmosphere. On the following day, serum samples were first diluted 1:10 with the modified diluent medium and then subjected to two-fold serial dilutions ranging from 1:20 to 1:20,480, with 40 µL per dilution well in duplicate. Virus stocks were prepared to achieve approximately 60 foci per well, based on prior back titration results. Equal volumes (40 µL) of diluted virus were mixed with the diluted serum samples, and the mixtures were incubated at 37 °C for 30 min. Concurrently, the culture medium was removed from the Vero plates. After incubation, 30 µL of each virus-serum mixture was transferred into the corresponding wells containing Vero cells and incubated at 37 °C for 1 h to allow virus adsorption, with gentle rocking of the plates after 30 min. Following adsorption, 150 µL of pre-warmed overlay medium was added to each well, and the plates were incubated at 37 °C in 5% CO_2_ for 4 days.

On day 6, the overlay medium was aspirated, and the wells were gently washed twice with 180 µL of 1× phosphate-buffered saline (PBS). Cells were then fixed with 100 µL of 80% methanol for 10 min at room temperature. Plates were either immediately processed for immunostaining or stored at −80 °C for subsequent analyses.

### Immunostaining and focus enumeration

Following methanol fixation, the 96-well plates were air-dried and washed twice with 180 μL of PBS, followed by gentle blotting to remove residual liquid. To block nonspecific binding, 180 μL of PBS containing 5% non-fat dry milk was added to each well and incubated at room temperature for 30 min. The blocking buffer was then removed, and 100 μL of primary antibody solution (1:2000 dilution of Anti-Dengue Virus Complex Antibody, MAB8075) was added to each well. Plates were incubated at room temperature for 2–3 h with gentle shaking at 30 rpm.

After primary antibody incubation, the wells were washed four times with 180 μL of PBST (PBS containing 0.05% Tween-20), followed by blot drying. Secondary antibody solution (100 μL per well, 1:3000 dilution of Anti-Mouse IgG [gamma] Antibody, Cat. #5220-0460) was then added, and plates were incubated at room temperature for 1 h with shaking at 30 rpm. Wells were subsequently washed six times with PBST and air-dried.

To visualize the foci, 60 μL of TrueBlue™ Peroxidase Substrate (SeraCare, Milford, MA, USA; Cat. #5510-0030) was added to each well and incubated for 20 min in the dark. The substrate was then removed, and the plates were air-dried. Immunostained foci were counted using an automated ImmunoSpot analyzer (AID), and viral titers were calculated accordingly.

### Antibody isotype enzyme-linked immunosorbent assay

The full spike trimer D614G or envelope protein from DENV serotype 2 (Sino Biological, Beijing, China) was immobilized at 0.2 mg/mL per well in PBS on a 96-well flat-bottom polystyrene microplate overnight. A blocking solution of 2.5% bovine serum albumin (BSA) in PBST (PBS with 0.05% Tween 20) was applied. All samples were diluted in PBST under identical conditions: 1:200 for anti-E IgG subclasses and 1:1000 for anti-S or anti-E IgM and IgA. Horseradish peroxidase (HRP)-conjugated anti-human secondary antibodies were used at a 1:5000 dilution in 2.5% BSA in PBST: IgG1, IgG2, IgG3, and IgG4 (SouthernBiotech, Birmingham, AL, USA); IgM (Sigma-Aldrich, St. Louis, MO, USA); IgA (Invitrogen, Waltham, MA, USA); and IgG (IgG H+L; Sigma-Aldrich). To establish standards for measuring antibody isotype concentrations, the following recombinant proteins were immobilized in carbonate buffer (0.1 M NaHCO_3_, pH 9.6) and serially diluted: IgM from serum (1:20,000, 2 mg/mL, Sigma-Aldrich), IgA from plasma (1:20,000, 1.44 mg/mL, Sigma-Aldrich), IgG1 heavy chain (1:1000, 0.25 mg/mL, Sino Biological, Beijing, China), IgG2 heavy chain (1:5000, 0.25 mg/mL, Sino Biological), IgG3 heavy chain (1:20,000, 0.25 mg/mL, Sino Biological), and IgG4 heavy chain (1:10,000, 0.25 mg/mL, Sino Biological). For IgG1 a full anti-envelope human monoclonal antibody was also used (Anti-human DENV EDE1 IgG1, Creative Biolabs, Shirley, NY, USA). For heavy chain standards, concentrations were adjusted to account for the lower molecular mass and the presence of two heavy chains in a complete IgG antibody.

For the determination of the nucleocapsid titer (n-titer) 96‐well flat bottom plates were coated with 2 µg/mL of nucleocapsid protein of SARS‐CoV‐2. Serum or plasma is diluted in 1:200, and antihuman IgG‐HRP (horseradish peroxidase) is used in combination with tetramethylbenzidine (TMB), which is stopped by 25% sulfuric acid. The optical density is then measured by a microplate reader. A value of 0.3 OD was used as cutoff for positive and negative cases.

### Anti-E Fc-receptor affinity assay

The envelope protein of DENV type 2 (Sino Biological) was immobilized at 0.2 mg/mL per well in a 96-well flat-bottom polystyrene microplate using bicarbonate buffer (NaHCO_3_, pH 9.6) and incubated overnight. A blocking solution of 2.5% BSA in TBST (TBS with 0.05% Tween 20) was applied to reduce nonspecific binding. After washing the plates with TBST, samples diluted 1:100 in TBST were added and incubated for 1 h. The plates were then washed four times with TBST, followed by the addition of biotinylated Fc-receptor CD64, CD16a or CD32a (AcroBiosystems, Newark, DE, USA) and streptavidin-alkaline phosphatase (Promega, Madison, WI, USA; diluted 1:10,000) in 2.5% BSA blocking solution for 1 h. The plates were then washed nine times, and 50 µL of highly sensitive Tn-Cyclon reagent (BioPhenoMA, Tokyo, Japan) was added for signal detection. Absorbance at 405 nm was measured kinetically over 1 h. The maximum reaction rate was used to quantify the signal, and the fold change was calculated relative to nonspecific IgG from human serum.

### Removal of anti-S or anti-E antibodies

BcMag™ Tosyl-Activated Magnetic Beads (BioClone, San Diego, CA, USA) were utilized to deplete anti-S or anti-E antibodies. For antigen coupling, 100 μg of full spike or envelope protein (Sino Biological) was conjugated to 30 mg of Tosyl magnetic beads. The beads were then evenly distributed across samples, with each sample receiving beads coupled with 2.5 μg of antigen. Serum or plasma samples were diluted at least 1:2000 in 600µl PBS, followed by incubation with over-the-top rotation at room temperature for 2 h. The supernatant was collected and analyzed via ELISA or ADE assays. Data were included only if the corresponding antibody titer was reduced by at least 80%. If the reduction was less than 80%, the experiment was repeated with higher sample dilutions (1:4000, 1:8000, or 1:10,000) until an 80% or greater reduction was achieved; otherwise, the data were excluded. The specificity of this method was confirmed using BSA-coated beads as a control, which resulted in a non-significant reduction in anti-S or anti-E antibody reactivity (9%, p = 0.06 and 24%, p = 0.06, respectively, **Supplementary Figures S3C, D**).

### ADE assay for serum antibodies

DENV type 2 was incubated at a multiplicity of infection (MOI) of 0.001 with serum diluted 1:1000 for 30 min on ice. The mixture was then incubated with 10 (5) THP-1 cells for 90 min. The THP-1 cells were washed twice, and the supernatant was collected after 48 h for quantitative PCR (qPCR).

### qPCR of Dengue 2 virus

Reverse transcription was performed using 5 μL of total RNA in 20 μL reactions with Reverse Transcription Mix (RT Enzyme & Kit; PURIGO Biotechnology Co., Ltd., Taiwan) following the manufacturer’s instructions. qPCR was conducted on a LightCycler^®^ 480 Instrument II (Roche, Basel, Switzerland) in 10 μL reactions using TaqMan™ Fast Advanced Master Mix (Applied Biosystems™, Waltham, MA,USA; catalog #4444556), approximately 1 μL of sample cDNA, and 10 μM each of forward and reverse primers (Dengue primers from Taiwan CDC). The qPCR protocol included pre-incubation at 95°C for 3 min, followed by 45 cycles of amplification (denaturation at 95°C for 10 s, annealing at 60°C for 20 s, and extension at 72°C for 5 s). After a final extension, a melting curve analysis was performed (95°C for 5 s, 60°C for 20 s, and cooling at 40°C for 30 s). All samples were run in technical duplicates, and expression levels were quantified as cycle quantification (Cq) values. For the qPCR analysis, the ΔΔCt method was applied, with gene expression fold changes expressed as log_2_ values.

### Antibody class depletion

For IgG or IgA depletion experiments, 50 µL of Protein G (for IgG, Fast Flow Cytiva) or IgA CaptureSelect affinity matrix (for IgA, Thermo Fisher) was loaded into a minispin column (BioVision) and washed twice with 600 µL of PBS. Each sample (5 µL) was diluted 1:20 and incubated in the column for 1 h with overhead rotation. The IgG- or IgA-depleted flow-through was collected through centrifugation at 100 × *g* for 1 min. The spin column was washed twice with 600 µL of PBS, and bound IgG or IgA was eluted twice with 600 µL of 0.1 M glycine (pH 2.7) and washed two times again in PBS. The IgG- or IgA-depleted flow-through was then reloaded onto in the column again and incubated overnight at 4°C with overhead rotation to remove any residual IgG or IgA. The flow-through was collected again and subjected to the ADE, ADCP, or ADCD assays. For IgM depletion, serum was diluted 1:10 in 10% polyethylene glycol 8000 in PBS and incubated for 30 min at 4°C, followed by centrifugation at 10,000 × *g* for 20 min at 4°C. The supernatant was collected, and IgM depletion was confirmed using anti-IgM ELISA. Because this method also resulted in significant IgG losses, the IgG concentration in the IgM-depleted samples was measured, and volumes were adjusted to match the IgG concentration of the original serum samples.

### IgG purification

IgG was purified using a Melon Gel (Thermo Fisher Scientific). The Melon Gel was packed into a minispin column and equilibrated through centrifugation (2000 × *g*) with Melon Gel Purification Buffer. Each sample (5 µL) was diluted 1:20 in the purification buffer and incubated on the column for 30 min with end-over-end rotation. Purified IgG was collected through centrifugation, and the samples were buffer-exchanged into PBS using Zeba Desalt Spin Columns.

### Antibody-dependent cellular phagocytosis assay and antibody dependent complement deposition assays

The ADCP and ADCD assays were adapted from our previous study (9). The DENV2 E protein was biotinylated using the Micro Sulfo-NHS-LC-Biotinylation Kit (Thermo Fisher Scientific). Beads (5 × 10^8^) were mixed with 100 ng DENV E2-biotinylated protein overnight. Serum, IgG purified, or IgG depleted samples were diluted to 1:100 as final dilution in FACS buffer, and endogenous complement was inactivated through incubation at 56°C for 30 min. The inactivated serum or plasma was then incubated with the beads at 37°C for 2 h to enable binding of antigenic antibodies to the beads. For the ADCP assay, the beads were washed twice with a medium containing monocyte-like THP-1 cells (Taiwan Food Industry Research and Development Institute) and then incubated with 1 × 10^5^ cells for 15–18 h. The cells were then washed using centrifugation at 400g for 3 min with PBS (2×) and incubated with trypsin EDTA (0.25%; Gibco, Waltham, MA, USA) for 10 min. The cells were washed again twice and resuspended in 800 µL of FACS buffer and subjected to flow cytometry (Thermo Attune NxT cell analysis). For the ADCD assay, the antibody complexes were incubated in 1/50 guinea pig complement (MP Biomedicals, Santa Ana, CA, USA; cat# 086428-CF) in RPMI 1640 medium for 15 min, followed by two washes with 15 mM EDTA in PBS. The beads were then incubated with an anti-goat C3 antibody (MP Biomedicals; cat# 0855367) for 15 min, followed by two washes, and incubation with an anti-goat fluorescent antibody (donkey anti-goat IgG H&L, AF647 Abcam cat# ab150135) for 15 min. Next, the beads were resuspended in 1 mL FACS buffer for flow cytometry. To measure ADCP, a phagoscore (10, 17)was calculated as the fraction of bead-positive cells multiplied by the mean fluorescence intensity (MFI). The MFI for detecting AF647 was used for ADCD.

### Complement inactivation

To inactivate complement, the serum was diluted 1:500 in RPMI medium and incubated at 56°C for 30 min. For the complement rescue experiment, guinea pig complement (MP Biomedicals, Santa Ana, CA, USA; catalog #086428-CF) was added at a 1:50 dilution following heat treatment. Previous studies have shown that guinea pig complement can interact with and be activated by human antibodies with similar efficiency (17).

### Statistical analysis

Statistical analyses were performed using R software (ver. 4.3.1) and the dplyr package. All figures were created using R. For violin plots, the median is given. The Mann–Whitney U test was used for comparisons between two samples. The pairwise Wilcoxon test was used to compare multiple groups. Values of p < 0.05 were considered significant. Pearson correlation coefficients were measured using the cor.test function in R to evaluate relationships between IgG profile parameters and other variables (age, Fc-affinity, ADE, and time interval from injection). To classify the cross-reactive antibody response, the mean and standard deviation of the differences between duplicate samples from the anti-E ELISA were calculated. The threshold for classification was defined as the mean plus the standard deviation (mean + standard deviation = threshold). If | untreated(sample x)−anti_S_removed(sample x)|<threshold, then there was no cross-reaction. If there was a significant cross-reaction, full cross-reaction was determined by anti_S_removed(sample x)−anti_E_removed(sample x)<threshold; otherwise, it was classified as a partial cross-reaction. The classification was validated by applying a k-means clustering algorithm (kmeans function in R) to the data, using three clusters corresponding to the three groups: no cross-reactivity, partial cross-reactivity, and full cross-reactivity.

## Results

### Anti-Dengue envelope antibodies by COVID-19 vaccination and DENV infection show distinct antibody isotype profiles

**Figure 1:**
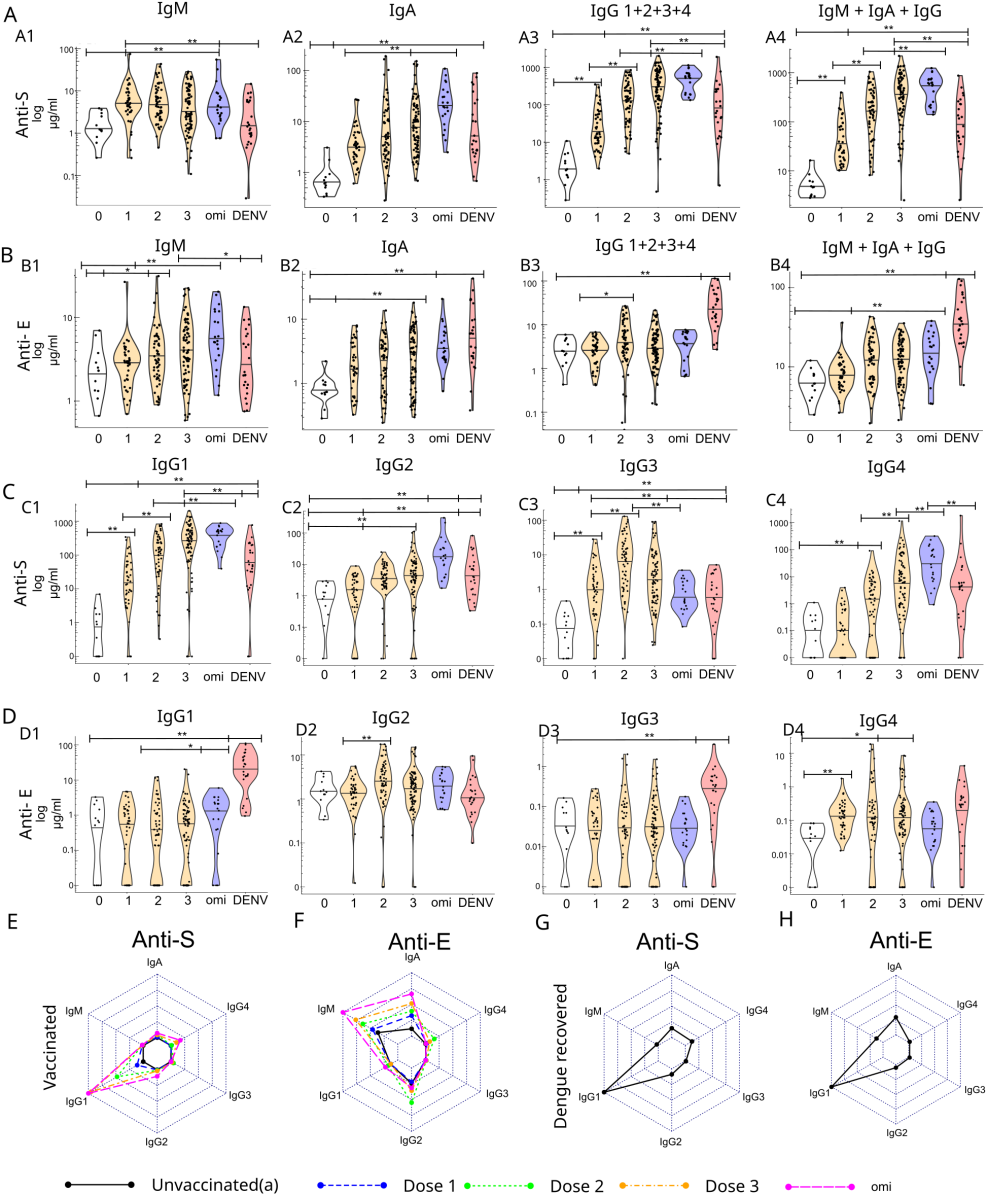
Antibody titers for anti-SARS-CoV-2 spike (anti-S) and anti-Dengue type 2 envelope (anti-E) were measured in unvaccinated, COVID-19-vaccinated, and dengue-recovered individuals. **(A, B)** Titers are reported in µg/mL for each isotype (IgM, IgA, total IgG [IgG1 + 2+3 + 4], and the sum of all isotypes) in sections. **(C, D)** Titers for anti-S and anti-E are shown for each IgG subclass (IgG1–4) in sections. The average contributions of each isotype to anti-S and anti-E responses in the COVID-19-vaccinated group **(E, F)** and for the dengue-recovered group are shown in radar plots **(G, H)**. Data in radar plots were normalized to the highest average isotype concentration. *p < 0.05 and **p < 0.01.

We collected 271 serum and plasma samples from a previous Taiwanese population-based study to assess the titers of anti-SARS-CoV-2 spike glycoprotein (anti-S) and anti-envelope (anti-E) IgM antibodies. The samples were categorized by vaccination status as follows: unvaccinated (0: n = 14), vaccinated with one to three doses of COVID-19 vaccines targeting the Wuhan variant (1: n = 44; 2: n = 60; 3: n = 90), including adenovector, mRNA, and protein subunit vaccine types (**Table 1**). Additionally, we collected samples from individuals who received an Omicron-targeted booster, resulting in a total of 4–7 COVID-19 vaccine doses (XBB and BA.4.5, omi, n = 21,**Supplementary Table S1**). We also collected samples from 24 individuals in southern Taiwan who had recovered from the 2023 dengue outbreak, with samples obtained at a median of 14 days post-diagnosis (range: 5–21 days). These infections were identified as either DENV serotype 1 or serotype 2 (**Supplementary Table S2**). Both anti-S and anti-E IgM antibody levels increased significantly after the first dose of the COVID-19 vaccine (**Figures 1A1, B1**). Anti-S and anti-E IgM titers were comparable in COVID-19 vaccinated, with the median anti-E titer reaching 81% of the median anti-S titer (median anti-S: 4.3 µg/mL; anti-E: 3.5 µg/mL). In dengue recovered, neither anti-S nor anti-E IgM titers were significantly elevated. For IgA antibodies, both anti-S and anti-E titers increased with each additional vaccine dose and were significantly higher than those in the unvaccinated cohort (**Figures 1A2, B2**). In individuals vaccinated against COVID-19, the median anti-E IgA antibody titer was 42.3% of the anti-S IgA titer (median anti-S: 5.9 µg/mL; median anti-E: 2.5 µg/mL). In individuals who recovered from dengue infection, the median anti-E IgA titer (5.9 µg/mL) was higher than that in COVID-19-vaccinated individuals. For IgG antibodies, anti-E IgG titers showed a slight but significant increase from the first to the second COVID-19 vaccine dose, but remained low (median 1.4%) compared with anti-S IgG titers (median anti-S: 182.6 µg/mL; median anti-E: 2.5 µg/mL), which increased with each subsequent dose (**Figures 1A3, B3**). In individuals who recovered from dengue infection, anti-E IgG antibody titers were significantly elevated, as expected (median: 22.1 µg/mL). In COVID-19-vaccinated individuals, both anti-S and anti-E antibody titers were elevated across IgM, IgA, and IgG isotypes. The anti-E antibody titers in COVID-19-vaccinated individuals were lower than those elicited by DENV infection (**Figures 1A4, B4**). Titers of all IgG subclasses specific to anti-S (IgG1, IgG2, IgG3, and IgG4) were significantly higher in COVID-19-vaccinated individuals than in the unvaccinated population. Additionally, anti-S IgG1, IgG2, and IgG4 titers increased with each subsequent vaccine dose, consistent with our previous findings (**Figure 1C**) (9). For anti-E IgG in the COVID-19-vaccinated group, anti-E IgG1 titers showed a significant increase in individuals who received the Omicron variant booster (**Figure 1D1**), IgG2 increased from the 1^st^ to the 2^nd^ dose, and IgG4 increased significantly after the 1^st^ and 3^rd^ doses (**Figures 1D2, D4**). Dengue-recovered individuals had a typical anti-viral anti-E IgG profile with significant increases in IgG1 and IgG3 (**Figures 1D1, D3**). Overall, our findings indicate that the anti-E antibody profile in COVID-19-vaccinated individuals was dominated by IgM and IgA isotypes, with titers increasing after each vaccine dose. In contrast, the anti-E antibody profile in dengue-recovered individuals and the anti-S antibody profile in vaccinated individuals were predominantly IgG1-driven (**Figures 1E–H**). No significant differences were observed in the anti-E antibody titers across IgM, IgA, and IgG isotypes among the different COVID-19 vaccine platforms (adenovector, mRNA, and protein subunit) in vaccinated individuals (**Supplementary Figure S1**). For anti-S antibody titers, IgA and IgG subclass titers were significantly lower in individuals vaccinated with the ChAdOx1 nCoV-19 adenovector vaccine than in those vaccinated with mRNA vaccines or the protein subunit vaccine MVC-COV1901, consistent with our previous findings (9). No significant correlations were observed between age and anti-S or anti-E antibody titers across IgM, IgA, and IgG isotypes, apart from anti-E IgG1, which showed a significant negative correlation with age following the first COVID-19 vaccine dose (**Supplementary Figure S2A**). The interval between COVID-19 vaccination and sample collection was positively correlated with anti-E IgG2 titers following the first vaccine dose. Conversely, after the Omicron-targeted booster, the interval between vaccination and sample collection was negatively correlated with both anti-E IgM and anti-E IgG2 titers (**Supplementary Figure S2B**). Sex had no significant influence on the antibody isotype titer (**Supplementary Figures S2C1–H2**).

### Anti-E and anti-spike titers are low before vaccination and vary significantly among ethnicities and regions

**Figure 2:**
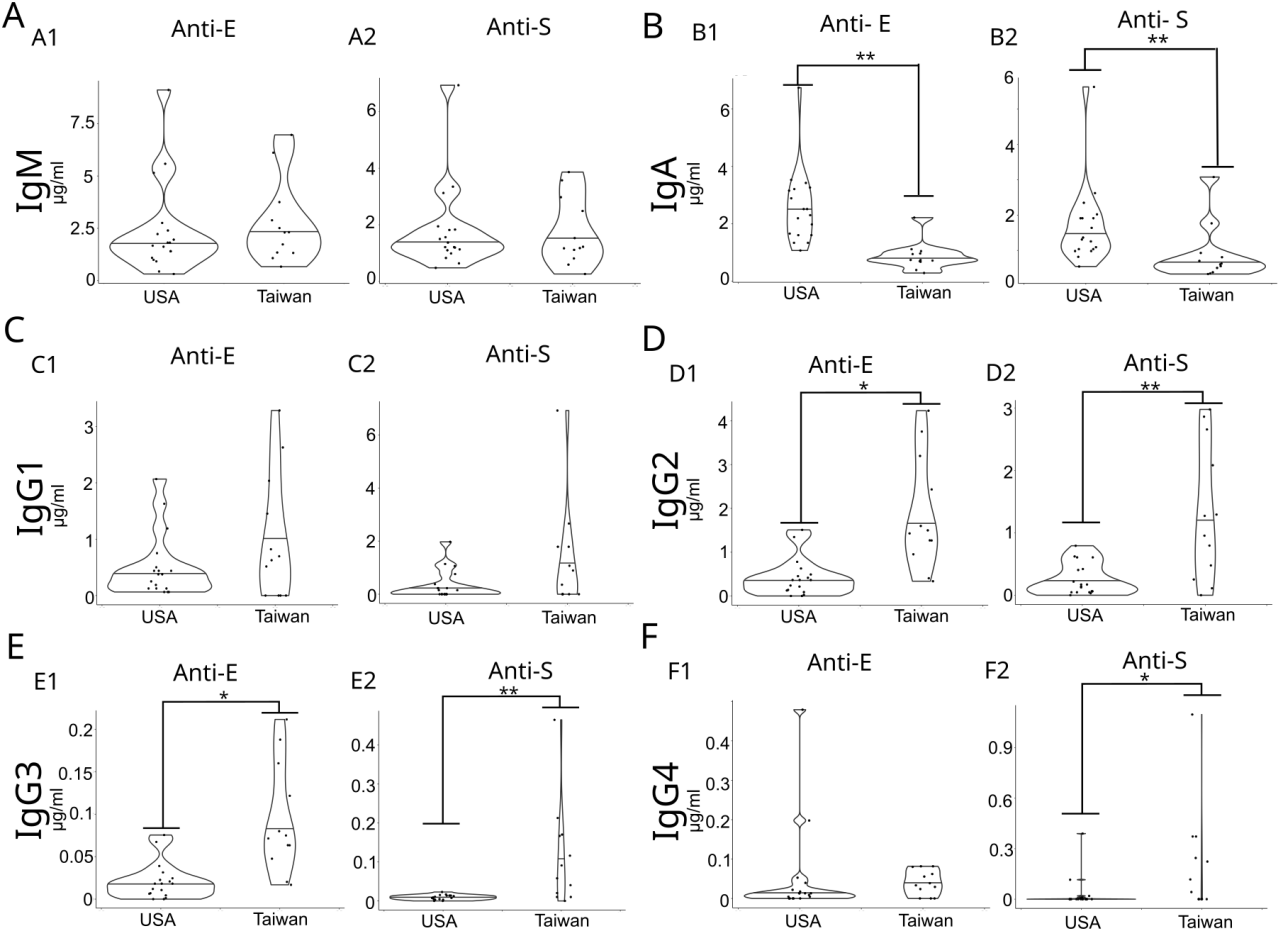
Concentrations of anti-E and anti-S antibodies were measured in non-vaccinated Taiwanese and pre-pandemic U.S. cohorts for the following isotypes: **(A)** IgM, **(B)** IgA, and **(C–F)** IgG subclasses. *p < 0.05, **p < 0.01.

We detected low levels of anti-S and anti-E antibodies in the unvaccinated Taiwanese cohort (**Figure 2**). Given that flaviviruses, such as DENV and Japanese encephalitis virus, are endemic in Taiwan and samples were collected after the onset of the COVID-19 pandemic, we investigated whether these low antibody levels were specific to the Taiwanese population by comparing anti-S and anti-E antibody titers with those in pre-pandemic samples from the United States (**Supplementary Table S3**). The United States samples exhibited detectable anti-S and anti-E titers. No significant differences were found in anti-S and anti-E IgM titers between the two cohorts (**Figure 2A**). The United States cohort showed significantly higher anti-S and anti-E IgA titers than those in Taiwan (**Figure 2B**), while the Taiwanese cohort had higher anti-E IgG2, IgG3 subclass levels, while for anti-S IgG2,3 and 4 was elevated in comparison to the US cohort (**Figures 2C–F**).

### Anti-E antibodies from COVID-19-vaccinated individuals are cross-reactive to the SARS-CoV-2 spike protein

**Figure 3:**
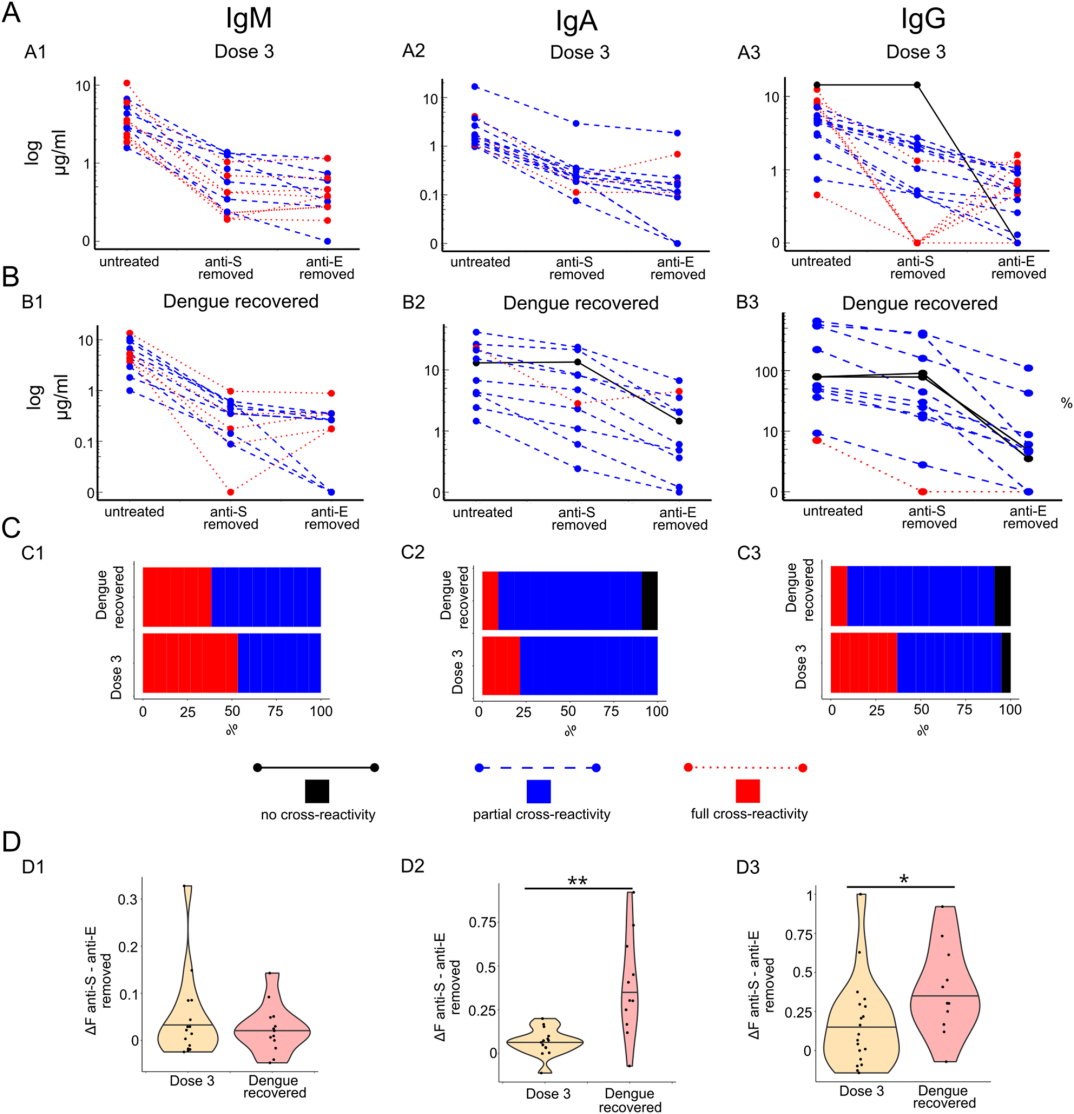
**(A, B)** Anti-E titer before (untreated) or after the removal of anti-S or anti-E antibodies with magnetic beads from COVID-19-vaccinated (three doses) or Dengue-recovered individuals for IgM, IgA, and IgG. Samples classified as non-cross reactive are marked in black with solid lines, partial cross-reactive blue with dashed lines, and full cross-reactive in red with dotted lines. **(C)** Distribution of non-, partial, and fully cross-reactive anti-E samples. **(D)** Level of cross-reactivity were estimated as $\frac{\text{"anti-S removed"}-"anti-E\;removed"}{untreated}$. *p < 0.05, **p < 0.01.

We investigated the cross-reactivity of anti-E and anti-S antibodies in the context of COVID-19. We initially analyzed the correlation between anti-E and anti-S antibody titers, observing the strongest and most significant correlations in the unvaccinated population for IgM, IgA, IgG1, and IgG2 (**Supplementary Figure S3A**). In contrast, the vaccinated population showed significantly weaker correlations, only for IgM and IgA (**Supplementary Figure S3B**). Among individuals who recovered from DENV infection, a significant correlation between anti-S and anti-E was observed solely for IgM (**Supplementary Figure S3C**). These findings suggest that cross-reactive antibodies are present prior to vaccination. To directly test whether elevated anti-E antibodies in COVID-19-vaccinated individuals cross-react with the SARS-CoV-2 spike protein, we selectively depleted either anti-S or anti-E antibodies using magnetic beads coated with S or E proteins, respectively. If anti-E antibodies are cross-reactive with anti-S antibodies, the depletion of anti-S antibodies would also reduce anti-E titers. No significant reduction was observed when using only BSA-coated beads (**Supplementary Figure S3D, E**), showing that the assay did not remove unspecific anti-E and anti-S. Our results confirmed cross-reactivity, as the depletion of anti-S antibodies led to a reduction in anti-E titers for IgM, IgA, and IgG isotypes (**Figure 3**). In all COVID-19-vaccinated individuals (third dose, n = 19) and dengue-recovered individuals (n = 12), we confirmed cross-reactivity for IgM antibodies in all investigated samples (**Figures 3A1-C1**). Furthermore, the depletion of anti-E reduced anti-S IgM titers significantly, to a comparable extent across all samples (**Supplementary Figures S3F, G**), indicating that most anti-S and anti-E IgM antibodies were cross-reactive. Cross-reactivity was observed for anti-E IgG and IgA antibodies (**Figures 3B1-C3**). Notably, one individual in the vaccinated cohort and two in the dengue-recovered cohort showed no detectable cross-reactive anti-E IgG antibodies, while one individual in the dengue-recovered cohort lacked cross-reactivity for IgA (**Figures 3C2, C3**). Depletion of anti-S antibodies significantly reduced anti-E IgG and IgA titers; however, depletion of anti-E antibodies did not significantly affect anti-S titers, suggesting that most anti-S antibodies induced by vaccination are not cross-reactive (**Supplementary Figures S3H–K**). The degree of cross-reactivity was quantified by calculating the relative difference between anti-E antibody titers after anti-S depletion and anti-E depletion, with 0 indicating complete cross-reactivity and 1 indicating no cross-reactivity. This analysis revealed that IgM cross-reactivity was similar between the COVID-19 vaccinated and dengue-recovered cohorts. In contrast, IgG and IgA cross-reactivity was higher in the COVID-19 vaccinated cohort than in the dengue-recovered cohort, suggesting that natural dengue infection induces antibodies targeting non-cross-reactive epitopes (**Figure 3D**).

### Cross reactive IgG antibodies from coronavirus (COVID)-19-vaccinated individuals can enhance DENV infection despite low Fc-affinity

**Figure 4:**
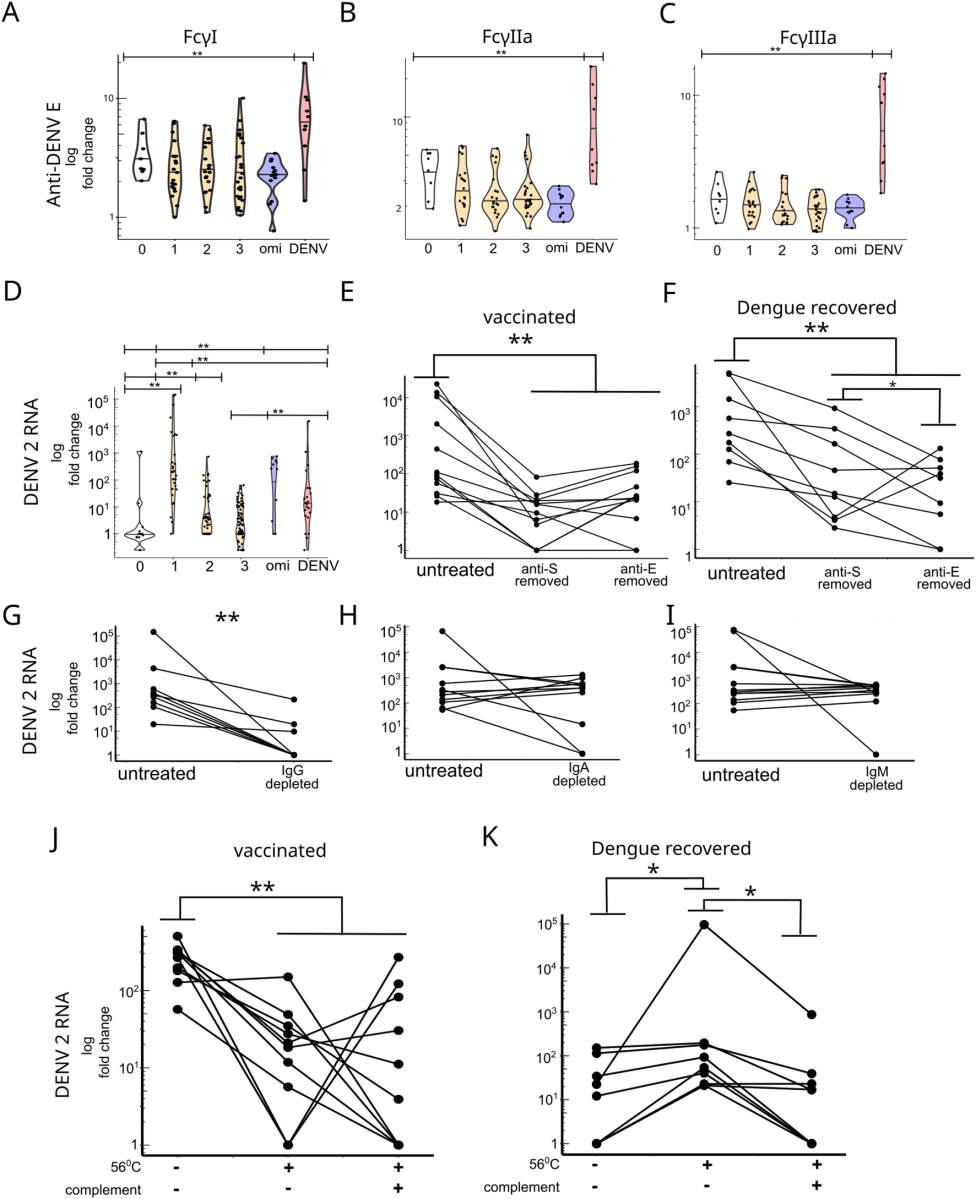
**(A–C)** Affinity of anti-E antibodies to FcγI, FcγIIa and FcγIIIa IgG receptors. Fold change values were measured as the ratio to the signal for IgG isotype unspecific for dengue. **(D)** ADE in THP-1 monocytes. Viral replication was measured using (qPCR) for dengue virus RNA. Fold change values were estimated relative to the virus-only treatment. **(E, F)** ADE measured using qPCR before and after the removal of anti-spike or anti-E antibodies in vaccinated and dengue recovered individuals. **(G–I)** ADE before and after IgG, IgA, and IgM depletion in vaccinated individuals. **(J, K)** ADE measured in untreated versus complement inactivated sera (56°C) and complement inactivated versus guinea pig complement-supplemented samples in individuals vaccinated against (one dose) or recovered from dengue. *p < 0.05, ** p < 0.01.

A critical factor influencing the risk of severe secondary disease is the affinity of IgG antibodies for the FcγRIIIa receptor (15). The binding affinities of anti-E antibodies to FcγI, FcγRIIa, and FcγRIIIa receptors were lower in COVID-19-vaccinated individuals than in dengue-recovered individuals (**Figures 4A–C**). No differences in FcγR binding affinity of anti-E antibodies were observed among vaccines, except for the BNT162b2 vaccine, which exhibited significantly higher FcγRIIIa affinity after the first dose (**Supplementary Figure S4A–C**). We evaluated the ability of cross-reactive antibodies to induce ADE during *in vitro* DENV infections. DENV was incubated with serum/plasma samples, and the enhancement of infection was assessed using human monocyte-like cells (THP-1). A significant increase in ADE was observed after the first vaccine dose, followed by a decrease after the third dose (**Figure 4D**). No significant differences in ADE were observed among the different vaccine platforms (**Supplementary Figure S4D**). In individuals who received the Omicron booster, a significant level of ADE was observed. Similarly, antibodies from dengue-recovered individuals exhibited significant ADE. No significant correlation was found between ADE and either antibody isotype or anti-E Fc receptor affinity in COVID-19-vaccinated or dengue-recovered individuals, except for a significant correlation between FcγRIIa and FcγRIIIa binding affinities (**Supplementary Figure S4E, F**). Depletion of either anti-S or anti-E antibodies significantly reduced ADE in COVID-19-vaccinated individuals to a similar degree (**Figure 4E**). In dengue-recovered individuals, anti-E antibody depletion resulted in a greater reduction in ADE than anti-S antibody depletion (**Figure 4F**). Several studies have shown that ADE is mediated by IgG antibodies (2). To investigate whether this mechanism also applies to cross-reactive antibodies in vaccinated individuals, we conducted selective antibody class-depletion experiments for IgG, IgA, and IgM. IgG depletion significantly reduced *in vitro* ADE across all tested samples (**Figure 4G**), whereas no significant changes were observed in IgA- or IgM-depleted samples (**Figures 4H, I**). Classical ADE uses the same pathways as Fc receptor-mediated phagocytosis of mononuclear cells. To explore this mechanism, we performed an antibody-dependent cellular phagocytosis (ADCP) assay for DENV E protein using THP-1 mononuclear cells treated with serum, IgG-purified, or IgG-depleted samples. A non-significant reduction in ADCP was observed in IgG-purified samples, whereas a significant reduction in ADCP was observed in IgG-depleted samples (**Supplementary Figure S4G**), supporting the role of IgG-dependent Fc receptor-mediated interaction involving the DENV2 E protein. Antibody-dependent complement deposition against the DENV2 was significantly reduced in both IgG-purified and IgG-depleted samples (**Supplementary Figure S4H**), indicating that other antibody classes or serum components induce complement activation against the DENV2 E-protein. Previous studies have revealed that the complement system can suppress ADE (12, 18). IgG1/3 exhibit stronger binding affinity for C1q than IgG2/4, enhancing classical complement pathway activation (19); therefore, sera from individuals who recovered from DENV infection, with high IgG1 and IgG3 anti-E antibody titers, may exhibit stronger complement-mediated inhibition of ADE than that of sera from COVID-19-vaccinated individuals with elevated anti-E IgG2 and IgG4 titers. We inactivated the complement system using heat treatment at 56°C for 30 min. Complement inactivation significantly increased ADE activity in sera from dengue-recovered individuals, whereas it significantly reduced ADE activity in sera from individuals vaccinated with a single dose of COVID-19 vaccine (**Figures 4J, K**). In addition, supplementation with active complement restored ADE activity in sera from dengue-recovered individuals, but had varied effects on ADE activity in sera from COVID-19-vaccinated individuals (**Figures 4J, K**). In 22.2% of the samples (n = 2), the addition of complement in the COVID-19-vaccinated group restored ADE activity, whereas it reduced ADE in 66.6% of samples (n = 6) and had no effect in 22.2% (n = 2).

### Individuals positive for anti-nucleocapsid antibodies exhibited higher anti-spike titers but not anti-envelope titers

Antibodies against the SARS-CoV-2 nucleocapsid protein (N-protein) serve as a specific marker of prior infection, as all vaccines used in Taiwan are exclusively spike (S)-protein-based. Anti-N titers were measured in a subset of samples collected after February 2022, given the absence of significant outbreaks in Taiwan prior to that time. N-titers were assessed in 81 samples (unvaccinated: n=2; three-dose primary series: n=38; Omicron booster: n=17; dengue-recovered: n=23). Anti-N positivity was determined using anti-N IgG ELISA (**Supplementary Figure S5A**). Positivity rates were 54%, 64.7%, and 52.2% in the three-dose, Omicron booster, and dengue-recovered cohorts, respectively. Antibody-dependent enhancement (ADE) capacity was altered only in Omicron booster recipients, with significantly higher ADE observed in anti-N-positive individuals (**Supplementary Figure S5B**). Anti-N titers correlated significantly only with anti-S IgG3 titers (**Supplementary Figure S5C**). No differences in anti-E antibody class titers were observed between anti-N-positive and anti-N-negative individuals (**Supplementary Figures S5D–I**). SARS-CoV-2 infection is expected to boost anti-S titers as part of the immune response. Consistent with this, anti-N-positive individuals showed significantly higher anti-S IgG1 and IgG2 titers in the three-dose cohort, and higher anti-S IgG1 and IgA titers in the dengue-recovered cohort (**Supplementary Figures S5 E2, F2, G2**).

## Discussion

Our research provides the first characterization of distinct antibody isotype profiles for cross-reactive antibodies between SARS-CoV-2 and DENV. Although previous studies have reported cross-reactivity of antibodies from infected or vaccinated individuals between these viruses, no prior work has specifically examined differences in the antibody isotype distribution in response to SARS-CoV-2 antigens or in COVID-19-vaccinated individuals. One of the earliest studies in individuals recovered from COVID-19 found that dengue anti-E IgM kits predominantly showed false-positive results, consistent with IgM being the main cross-reactive antibody class we identified among cross-reactive anti-DENV antibodies (5). Additionally, few studies have explored epitope specificity for cross-reactive antibodies between SARS-CoV-2 and DENV. One study identified 24 potential IgA epitopes on the SARS-CoV-2 spike protein using *in silico* sequence comparisons (20). However, the referenced study did not provide direct evidence for the presence of cross-reactive antibodies and failed to detect cross-reactivity for some proposed peptide epitopes in dengue-recovered patients. Several studies have explored the isotype profiles of cross-reactive antibodies between DENV and other flaviviruses. For instance, a study of antibody responses in patients with DENV and Japanese encephalitis virus found no significant differences in the distribution of IgM, IgA, and IgG isotypes between the two viruses (21). A study of Zika-infected individuals identified a significant role for IgM cross-reactive antibodies in virus neutralization, with one individual exhibiting a notable contribution of IgA to neutralization (22). Another study focusing solely on DENV reported that each IgG subclass targets distinct domains of the DENV E-protein following prolonged seroconvalescence (23). Our study indicates that anti-dengue antibodies observed in vaccinated individuals without prior dengue infection are triggered by vaccination. This is supported by a clear dose-dependent effect, and anti-nucleocapsid (anti-N) positivity—a marker of natural SARS-CoV-2 infection—did not influence anti-DENV envelope (E) titers.

Cross-reactive antibodies are present in unvaccinated cohorts from both the United States and Taiwan. One possible explanation is that these antibodies originate from natural antibodies expressed without prior antigenic stimulation, although their levels may increase following antigenic exposure (24). Natural antibodies predominantly belong to the IgM class, and most anti-E and anti-S IgM antibodies exhibit cross-reactivity across all cohorts in our study. A study has shown that DENV infection activates natural IgM, IgA, and IgG antibodies and induces antibodies against unrelated viruses, such as poliovirus (25). Further investigation of B cells producing anti-E and anti-S antibodies is needed to evaluate the hypothesis. We could not determine the reasons for differences in cross-reactive antibody profiles between unvaccinated Asian Taiwanese and predominantly African American United States cohorts. Beyond genetic factors, environmental factors, such as prior antigenic exposure, may contribute; for example, childhood Japanese encephalitis vaccination in Taiwan (26) could account for the elevated anti-E IgG titers in the Taiwanese population (27) and studies from Thailand and Nepal have shown, that anti-JEV immunity is associated with a higher risk of severe dengue (28, 29).

Pre-existing antibody-secreting B-cells or prior antigenic exposure could account for the lack of significant antibody class switching, despite notable class switching to IgG2 and IgG4 in anti-S antibodies, as observed in previous studiesy (9, 11). Instead IgG2 was already the dominant anti-E IgG subclass in COVID-19 unvaccinated individuals. SARS-CoV-2 infection also diminishes the class switching of anti-S antibodies to IgG4 (30, 31). However, the precise mechanisms driving this dynamic remain unclear. Future research could investigate which epitopes are more resistant to antibody class switching to support the development of stable immune responses for immunotherapies.

We did not investigate the epitopes of cross-reactive antibodies or whether epitope specificity varies across different antibody isotypes. Several studies have already identified potential binding sites between SARS-CoV-2 spike and dengue envelope and non-structural proteins. Future studies should determine which of these sites are targeted by cross-reactive antibodies from COVID-19-vaccinated individuals and whether antibody class distribution varies across different epitopes (5, 7, 8). One study using sera from individuals recovered from COVID-19 identified an epitope on the E protein via phage display. A competitive ELISA demonstrated partial inhibition of antibody binding in sera from COVID-19-recovered individuals in Taiwan (6). Another study, also using sera from COVID-19 vaccinated individuals, identified a distinct, shorter epitope that may partially account for the observed cross-reactivity (8). Studies of HIV and bacterial pathogens have shown that the constant region of distinct IgG isotypes can affect the conformation of the Fab variable region, thereby influencing epitope specificity (32–34). Studies of autoimmune diseases and allergies have shown that different antibody isotypes target distinct epitopes (35–37). The cross-reactive epitope discovered by Cheng et al. (6) contains a glycosylation site, and the predominant cross-reactive antibodies in our study were IgM, with IgG2 as the primary IgG subclass. Both IgM and IgG2 can be produced through T-cell-independent mechanisms and may target glycan epitopes. Future research may include peptide-based epitope mapping and analyses of glycopeptide targets to further elucidate cross-reactivity.

Our results confirmed that cross-reactive antibodies induce ADE *in vitro* that is IgG dependent like ADE induced by natural dengue infection, despite their lower titers and Fc receptor affinity compared with those of antibodies in dengue-recovered individuals. This finding may be partly explained by differences in complement system involvement. Complement components C1q and C3 can inhibit ADE. As expected, dengue-recovered individuals exhibited lower ADE due to higher levels of anti-E IgG1 and IgG3, which have greater affinity for C1q than those of IgG2 and IgG4, leading to ADE inhibition either directly via C1q or through C3 activation (12, 18); this can trigger direct lysis or subject the virion to another phagocytosis pathway via C3R receptor. Unexpectedly, complement inactivation reduced ADE activity in SARS-CoV-2-vaccinated individuals, warranting further analyses of the role of the complement system in low-titer anti-dengue IgG2 and IgG4 antibodies or the involvement of other temperature-sensitive serum proteins. For the whole dengue virus there are also other targets like the prM can also be an epitope for enhancing antibodies and thus the anti-E titer may not represent all anti-Dengue antibodies that cause ADE (38).However, our results show a clear significant contribution of anti-E antibodies in ADE (**Figures 4E, G**). Different glycosylation between the recombinant E protein and the E-protein of the virus could lead to difference in the antibodies binding to the virus or in the anti-E ELISA (24). While the 1^st^ dose and Omicron-targeted booster caused a larger *in vitro* ADE than that in dengue-recovered individuals, an association with COVID-19 vaccination and dengue disease has not been reported. The observed effects may be explained by low IgG1 titers and reduced FcγRIIIa affinity, as low-fucosylated IgG1 with high FcγRIIIa affinity has been associated with severe secondary dengue disease in observational and animal studies (3, 15, 16). Therefore, these cross-reactive low FcγRIIIa affinity antibodies induced by COVID-19 vaccines may not be a risk factor for more severe dengue disease.

Previous studies have suggested that the Omicron variant or Omicron-specific booster reduces titers of cross-reactive anti-dengue antibodies for dengue virus serotype 2 (39). However, in our study, the Omicron-boosted cohort had the highest anti-E antibody titers among all vaccinated cohorts, accompanied by elevated ADE activity, which was further elevated in individuals which were anti-SARS-CoV-2 n-titer positive. Additionally, Omicron-boosted individuals showed increased anti-E IgG1 levels. A negative correlation was observed between the time interval from Omicron booster administration to sample collection and anti-E titers, suggesting a potential negative effect of the Omicron booster on cross-reactive antibodies in the long term. Given the diverse vaccination histories of Omicron-era booster recipients in our cohort, a study using paired samples collected before and after booster administration would be valuable. Furthermore, studies of the long-term impact of the Omicron-targeted booster on cross-reactive antibodies are necessary.

## Conclusion

Our study demonstrated that cross-reactive antibodies against DENV, induced by SARS-CoV-2 vaccination, exhibit a distinct IgM- and IgA-dominated isotype profile. Despite low anti-dengue IgG titers, these antibodies induced *in vitro* ADE. The mechanisms underlying these effects remain unclear and warrant further investigation. This is critical for future vaccine antigen design, as it requires careful consideration of the potential beneficial or detrimental effects of cross-reactive antibodies against other viruses.

## Limitations

In this study, the time interval between sample collection and vaccination was not fixed, resulting in variable sampling times. No significant differences were observed between groups with respect to age or sex distribution; however, data on comorbidities were not recorded. For Omicron-booster-vaccinated individuals, prior vaccination histories were inconsistent, with some participants unable to provide complete COVID-19 vaccination records. Our dengue-recovered cohort, collected in 2023, reflects a Taiwanese population where most individuals were either vaccinated against or exposed to SARS-CoV-2; data on cross-reactive antibodies in dengue-recovered individuals who were unexposed to SARS-CoV-2 were not available. In the antibody removal assay for anti-S and anti-E antibodies, samples with very low anti-E or anti-S titers were excluded owing to the high dilution required; however, a risk of incomplete antibody removal for high-titer samples remains, potentially introducing bias toward samples with specific titer ranges.

## Data Availability

The raw data supporting the conclusions of this article will be made available by the authors, without undue reservation.
