# Supplementary material for: COVID-19 vaccination induces cross-reactive dengue virus antibodies with altered isotype profiles and in vitro antibody-dependent enhancement
Source: Front Immunol. 2025 Dec 17;16:1683070. doi: 10.3389/fimmu.2025.1683070 (PMC12753992; doi:10.3389/fimmu.2025.1683070)
Supplement: Supplementary file 1 [file DataSheet1.pdf]

Supplemental material

COVID-19 Vaccination Induces Cross-Reactive Dengue Antibodies with Altered Isotype Profiles and In Vitro ADE

Sebastian Reinig[1], Chin Kuo[1], Sheng-Yu Huang[1], Kuei-Ching Hsiung[1,2], Po-Kai Chen[3], Etsuro Ito[3,4,5], Ing-Kit Lee[6,7], Ching-Yen Tsai[8], Shu-Min Lin[9,10], Shin-Ru Shih[1,2]

[1] Research center for Emerging viral infections, Chang Gung University, Taoyuan, Taiwan

[2] Biomedical Translation Research Center & Institute of Biomedical Sciences, Academia Sinica, Taiwan

[3] Department of Biology, Waseda University, Tokyo, Japan

[4] Graduate Institute of Medicine, Kaohsiung Medical University, Kaohsiung, Taiwan

[5] R&D Department, BioPhenoMA Inc., Tokyo, Japan

[6] Division of Infectious Diseases, Department of Internal Medicine, Kaohsiung Chang Gung Memorial Hospital, Kaohsiung, Taiwan

[7] College of Medicine, Chang Gung University, Taoyuan, Taiwan

[8] Division of Infectious Disease, Department of Internal Medicine, Kaohsiung Municipal Feng-Shan Hospital, Kaohsiung, Taiwan

[9] Department of Thoracic Medicine, Chang Gung Memorial Hospital, Chang Gung University, School of Medicine, Taoyuan, Taiwan,

[10] Department of Respiratory Therapy, Chang Gung Memorial Hospital, Chang Gung University, School of Medicine, Taoyuan, Taiwan

Table S1: Booster vaccines and prior vaccination history of the samples of the omicron targeted cohort. ChAdOx1 nCoV-19 (AstraZeneca, AZ), MVC-COV1901 (Medigen), or the mRNA vaccines mRNA-1273 (Moderna), BNT162b2 (BNT). Peptide: Experimental peptide vaccine from United Biomed, Protein subunit Novavax: NVX-CoV2373

Omicron booster	dose	Prior vaccinated
Novavax (XBB)	5	AZ-AZ-BNT-unknown
	5	AZ-AZ-Moderna-unknown
	5	Moderna-Moderna-unknown-unknown
	4	Unknown
	4	AZ-AZ-BNT

	7	United Biomed-United Biomed-Medigen-Medigen-BNT-BNT
	5	Moderna-Moderna-Medigen-Novavax
	7	Medigen-Medigen-Medigen-Medigen-Moderna-Moderna
	8	Peptide-Peptide-unknown
	5	AZ-AZ-BNT-unknown
	5	AZ-AZ-Medigen-unknown
	5	AZ-AZ-Moderna-Novavax
	5	unknown
Moderna (XBB)	5	AZ-AZ-Moderna-unknown
	4	BNT-BNT-BNT
	5	AZ-AZ-Moderna-Moderna(BA.4.5)
	6	Medigen-Medigen-BNT-Moderna(BA.1)-Moderna(BA.4.5)
	5	AZ-AZ-Moderna-Moderna(BA.1)
Moderna (Ba.4.5.)	4	AZ-AZ-Moderna
	4	BNT-BNT-BNT

Table S2: Details of the Dengue recovered cohort. ChAdOx1 nCoV-19 (AstraZeneca, AZ), MVC-COV1901 (Medigen), or the mRNA vaccines mRNA-1273 (Moderna), BNT162b2 (BNT). ND: Not determined

ID	age	sex	Interval (days from diag-nosis)	serotype	Number of symptoms	Prior COVID-19 vaccination	COVID infected
2	41-45	F	13	1	7	ND	no
3	31-35	F	18	1	7	ND	yes
4	61-65	M	15	ND	7	ND	yes
5	81-85	F	17	1	5	ND	no
6	76-80	F	20	1	8 (bleeding gum)	ND	no
7	66-70	M	9	1	8	Moderna-Moderna-Moderna	no
10	36-40	M	10	1,2	ND	ND	yes
11	21-25	F	18	1	ND	ND	yes
12	61-65	M	12	1	ND	AZ-AZ-Moderna-Moderna	no
13	66-70	M	18	ND	ND	Moderna-Moderna	yes
14	66-70	M	18	1	ND	ND	yes
15	81-	F	16	1	4	ND	no

	85						
16	21-25	M	14	ND	ND	BNT-BNT-Moderna-Moderna	no
17	41-45	F	12	ND	ND	BNT-BNT	no
18	51-55	M	10	2	4	ND	no
19	66-70	M	12	ND	ND	AZ-AZ-Moderna-Moderna	no
20	56-60	F	21	2	ND	ND	yes
21	56-60	F	12	1	ND	ND	yes
22	21-25	M	13	ND	ND	Medigen	yes
23	46-50	F	14	ND	ND	ND	no
28	51-55	M	5	ND	ND	Moderna-Moderna-Moderna	yes

Table S3: Details of the unvaccinated Taiwanese cohort and the Pre-pandemic US cohort.

	Pre-pandemic (USA)	Non-vaccinated (Taiwan)
n	18	14
Collection period	2017/4/28-2018/11/9	2021/8/19-2023-2022/6/7
age	39.05 (22-64)	37.2 (21-59)
Female(%)	66.6	71.4
Ethnicity	Black American (16), White (1), Hispanic (1)	Taiwanese (Chinese or Aboriginal, no detailed documentation about exact ethnicity) (14)

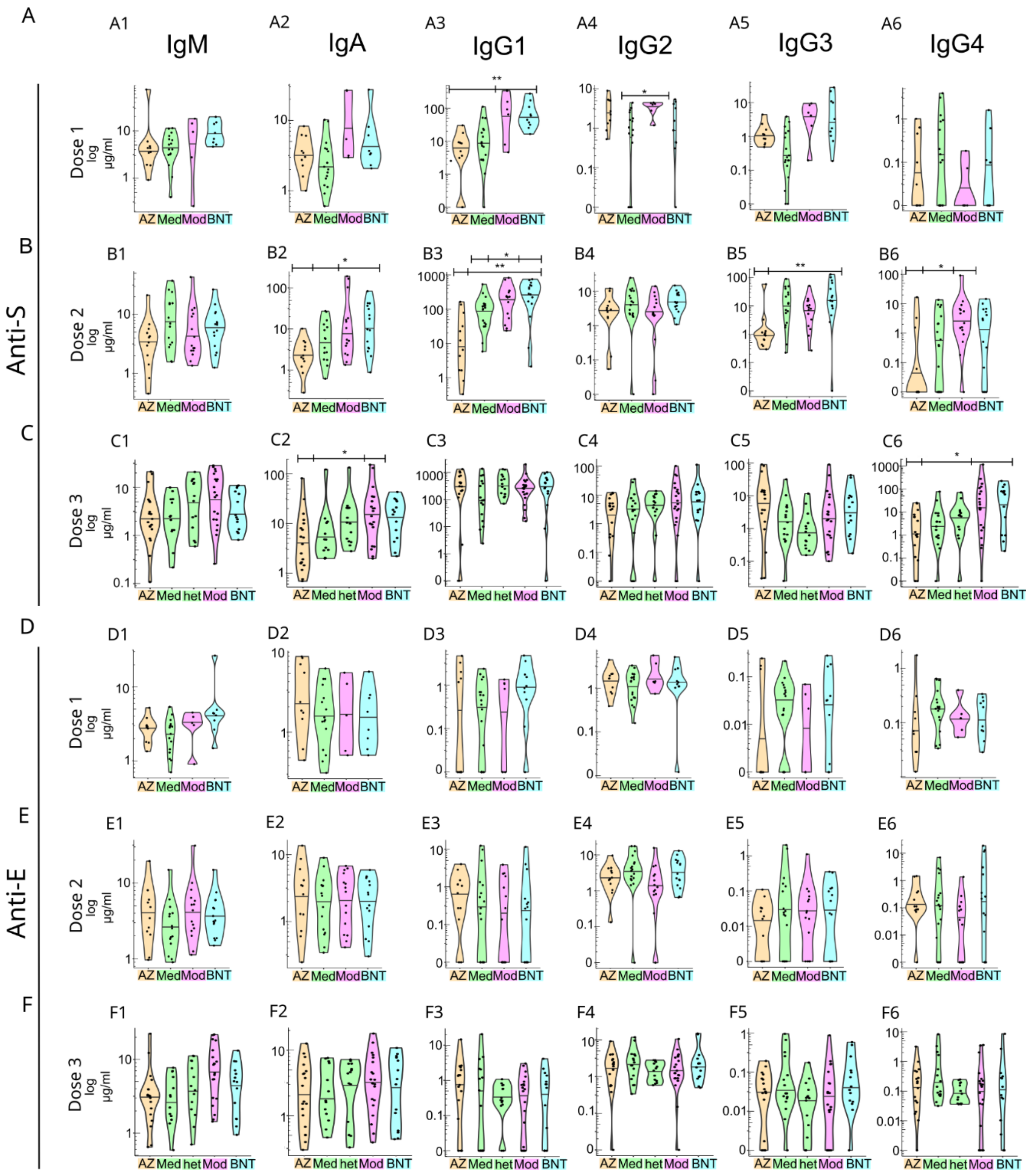

Figure S1: (A-C) anti-SARS-CoV-2 spike (anti-spike) antibody titer or (**D-F**) anti-Dengue type 2 envelope (Anti-E) titer between individuals vaccinated with different vaccine platforms for each Isotype (IgM,A IgG1-4). The antibody titer is given in µg/ml for each Isotype. * $p<0.05$, ** $p<0.01$, ChAdOx1 nCoV-19 (AstraZeneca, AZ), MVC-COV1901 (Med), or the mRNA vaccines mRNA-1273 (Mod), BNT162b2 (BNT). het: heterology (1-2nd dose non-mRNA and 3rd dose mRNA vaccine).

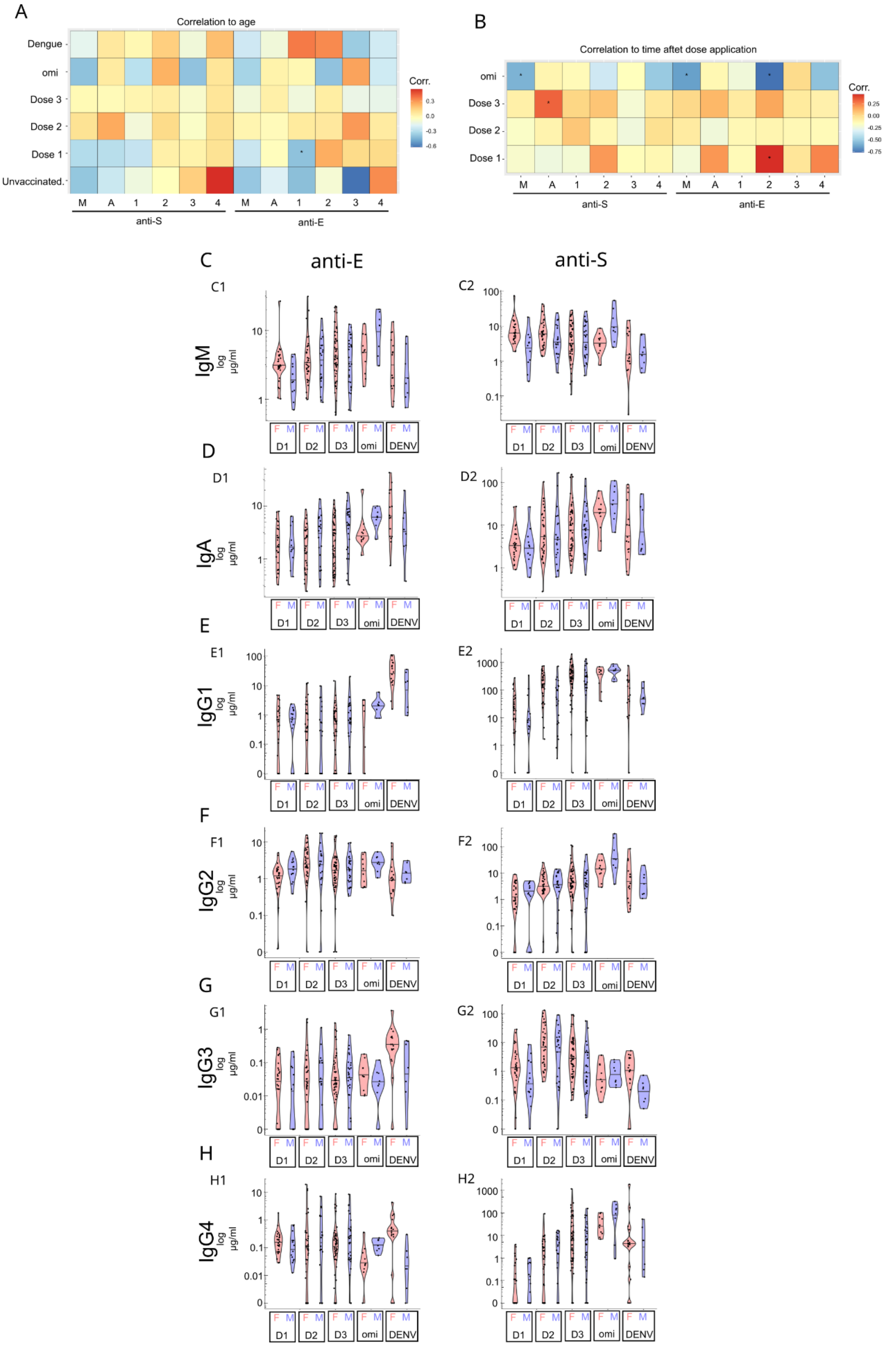
A
Correlation to age
Dengue
omi
Dose 3
Dose 2
Dose 1
Unvaccinated
M A 1 2 3 4 M A 1 2 3 4
anti-S
anti-E
Corr.
0.3
0.0
-0.3
-0.6
B
Correlation to time aftet dose application
omi
Dose 3
Dose 2
Dose 1
M A 1 2 3 4 M A 1 2 3 4
anti-S
anti-E
Corr.
0.25
0.00
-0.25
-0.50
-0.75
C
anti-E
anti-S
C1
C2
IgM log µg/ml
D
D1
D2
IgA log µg/ml
E
E1
E2
IgG1 log µg/ml
F
F1
F2
IgG2 log µg/ml
G
G1
G2
IgG3 log µg/ml
H
H1
H2
IgG4 log µg/ml
F M
D1 D2 D3 omi DENV

Figure S2: (A) Pearson correlation of antibody isotypes and cohorts to age or **(B)** the time interval after the last vaccine dose. (**C-H**) anti-S and anti-E Isotype titer separated by sex (F: Female and M: Male). * $p<0.05$, ** $p<0.01$

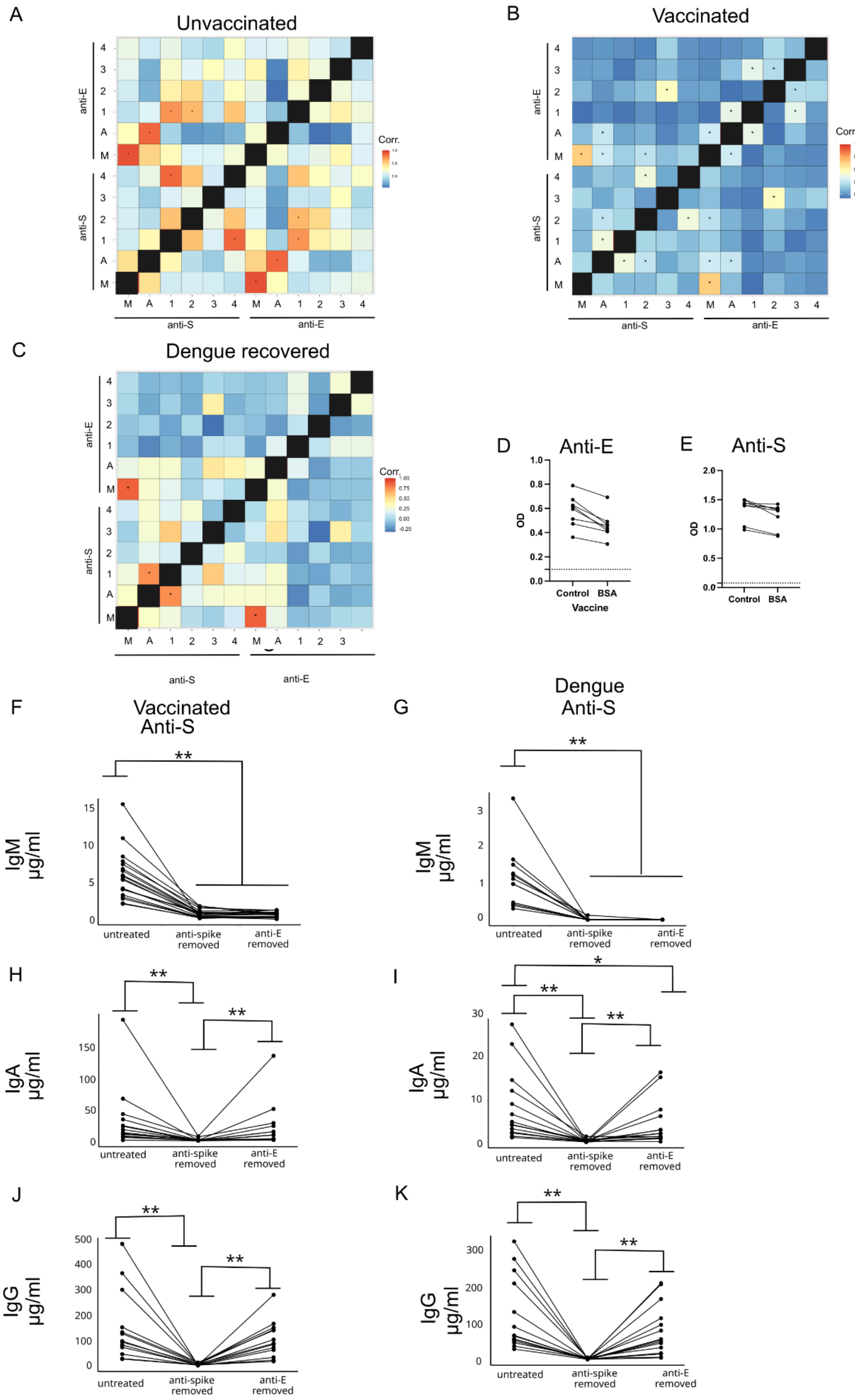
A
Unvaccinated
anti-E
anti-S
Corr.
M A 1 2 3 4 M A 1 2 3 4
B
Vaccinated
anti-E
anti-S
Corr.
1.00
0.75
0.50
0.25
0.00
C
Dengue recovered
anti-E
anti-S
Corr.
1.00
0.75
0.50
0.25
0.00
-0.25
D
Anti-E
OD
Control
BSA
Vaccine
E
Anti-S
OD
Control
BSA
F
Vaccinated
Anti-S
IgM
µg/ml
untreated
anti-spike removed
anti-E removed
G
Dengue
Anti-S
IgM
µg/ml
H
IgA
µg/ml
I
IgA
µg/ml
J
IgG
µg/ml
K
IgG
µg/ml

Figure S3: Pearson correlation between anti-S and anti-E of all (**A**) unvaccinated, (**B**) vaccinated, and (**C**) dengue recovered individuals between different isotypes. **(D)** Anti-S and (**E)** Anti-E IgG titer of beads before and after treatment with BSA coated beads to test specificity of antigen removal of antibodies. (**F-K**) anti-S titer before and after removal of anti-S or anti-E antibodies with magnetic beads either from COVID-19 vaccinated (3 dose) or Dengue recovered individuals. * $p<0.05$, ** $p<0.01$

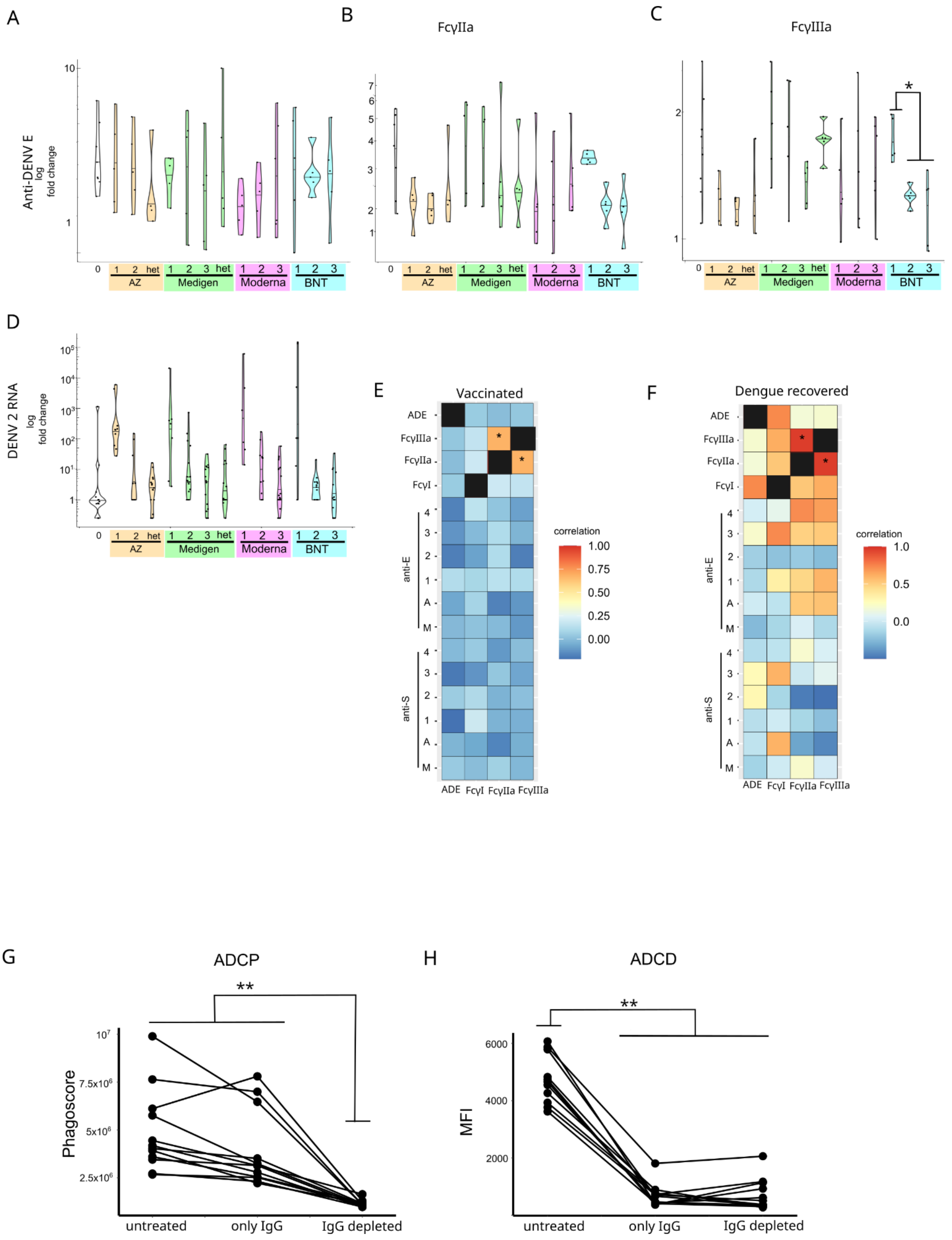

Figure S4: **(A-C)** Affinity of anti-E antibodies towards FcγI, FcγIIa and FcγIIIa IgG receptor for individuals receiving different vaccine platforms. Fold change is measured against IgG isotype

unspecific for Dengue. **(D)** Antibody dependent enhancement in THP-1 monocytes for each vaccine platform. Viral replication is measured by qPCR for Dengue RNA. Fold change is the ratio to virus only treatment. (**E,F**) Pearson correlation coefficients for ADE, FcγI, FcγIIa, and FcγIIIa to anti-spike or anti-E antibody isotypes. (**G,H**) Antibody dependent cellular phagocytosis (ADCP) and antibody dependent complement deposition (ADCD) against the E-protein measured with E-protein coated neutravidin beads for sera of COVID-19 vaccinated individuals. * p<0.05, ** p<0.01, ChAdOx1 nCoV-19 (AstraZeneca, AZ), MVC-COV1901 (Medigen), or the mRNA vaccines mRNA-1273 (Moderna), BNT162b2 (BNT). het: heterology (1-2nd dose non-mRNA and 3rd dose mRNA vaccine).

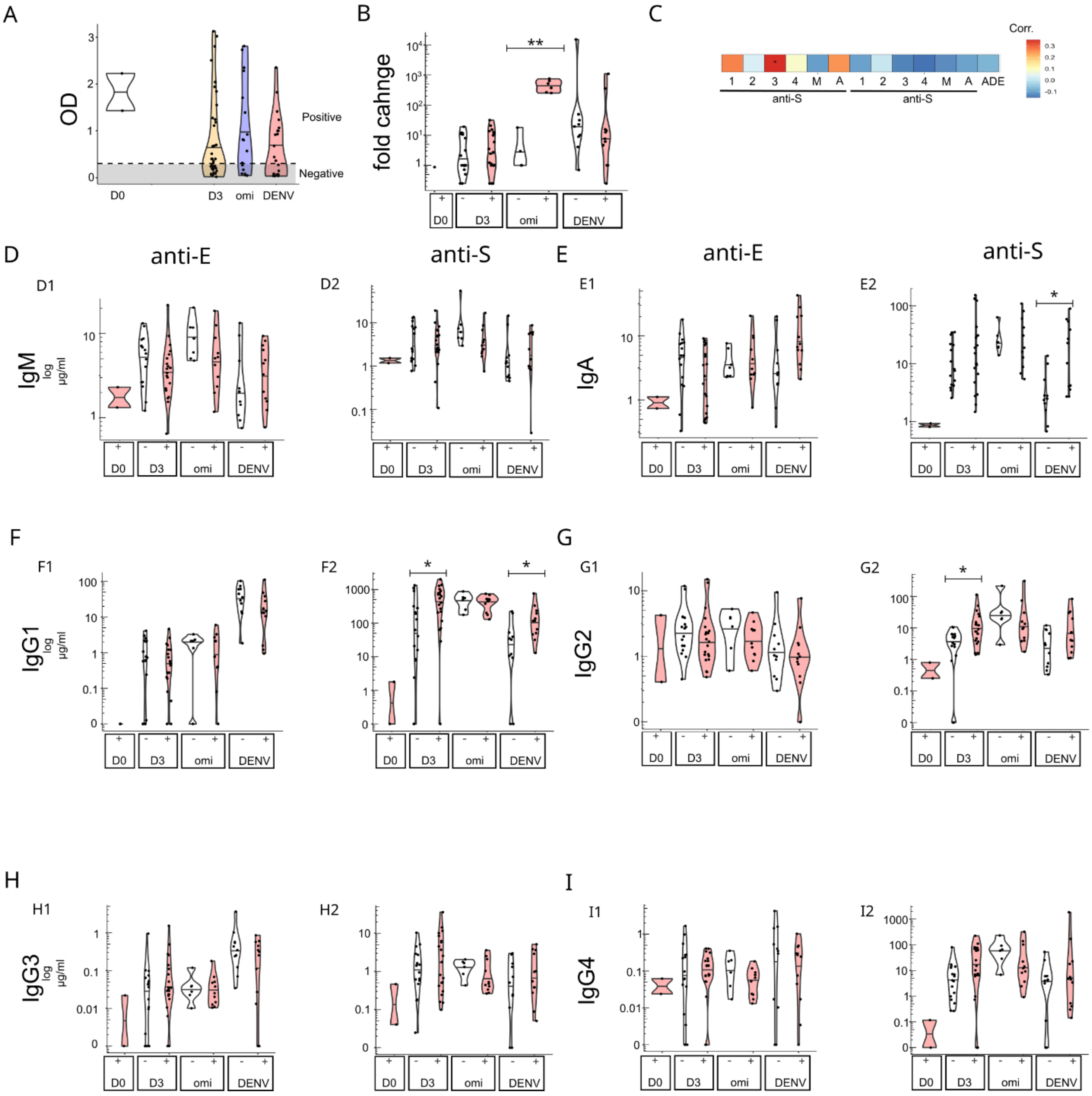

Figure S5: **(A)** N-titer for 81 samples in optical density (OD). An OD>0.3 was used as cutoff value for n-titer positive indicating prior SARS-CoV-2 infection. **(B)** ADE for each subgroup for n-titer negative and positive cases. (**C**) Pearson correlation coefficients for the n-titer relation for the antibody classes and ADE. (**D-I**) anti-S and anti-E antibody class titer separated by n-titer positive and negative samples. * $p<0.05$, D0: unvaccinated, D3: Dose 3 COVID-19 vaccinated, omi: omicron era booster, DENV: Dengue recovered. - : n-titer negative, +: n-titer positive
